# Depressive symptoms and cytokine levels in Serum and Tumor Tissue in patients with an Astrocytoma: a pilot study

**DOI:** 10.1186/1756-0500-7-423

**Published:** 2014-07-04

**Authors:** Angela R Starkweather, Paula Sherwood, Debra E Lyon, Dana H Bovbjerg, William C Broaddus, R K Elswick, Jamie Sturgill

**Affiliations:** 1 Department of Adult Health and Nursing Systems, Virginia Commonwealth University School of Nursing, 1100 East Leigh Street, P. O. Box 980567, Richmond, VA 23298, USA; 2 School of Nursing, School of Medicine, University of Pittsburgh Cancer Institute, University of Pittsburgh, 336 Victoria Building, 3500 Victoria Street, Pittsburgh, Pennsylvania 15261, USA; 3 College of Nursing, University of Florida, 1225 Center Drive, Gainesville, FL 32611, USA; 4 School of Medicine, Graduate School of Public Health, School of Arts and Sciences, University of Pittsburgh Cancer Institute, University of Pittsburgh, Hillman Cancer Center, Cooper Pavilion, Suite 140, Pittsburgh, Pennsylvania, USA; 5 Department of Neurosurgery, Virginia Commonwealth University Health Systems, Richmond, Virginia, USA; 6 Virginia Commonwealth University School of Nursing, 1100 East Leigh Street, P. O. Box 980567, Richmond, VA 23298, USA

## Abstract

**Background:**

Preoperative depressive symptoms are associated with poor outcomes in patients with an astrocytoma. Cytokines are associated with depressive symptoms in the general population and are important mediators of tumor growth and progression.

The aims of this study were to: (1) characterize depressive symptoms, other treatment-related symptoms and biological mediators; and (2) determine whether preoperative depressive symptoms were associated with the selected biological mediators.

**Methods:**

A prospective, exploratory study was carried out among 22 patients with a high-grade astrocytoma. Self-report questionnaires and peripheral blood samples were collected on the day of surgery. Tumor tissue was collected intraoperatively. Self-report questionnaires were assessed at 3, 6, 9, and 12-months postoperatively.

**Results:**

In circulation, serum IL-8 was inversely correlated with depressive symptoms while IL-17 measured in tumor tissue supernatant was inversely correlated with depressive symptoms. Depressive symptoms showed a significant increase at 12 months from baseline levels and were positively associated with treatment-related symptoms at 3 months and symptom distress at 12 months post-surgery.

**Conclusions:**

In this pilot study, depressive symptoms were negatively associated with IL-8 in serum and IL-17 in tumor tissue. The changes among depressive symptoms, treatment-related symptoms and symptom distress highlight the need for multi-faceted symptom management strategies over the treatment trajectory in this patient population.

## Background

It is estimated that 24,620 people will be diagnosed with a primary malignant brain or central nervous system tumor this year in the United States [1]. Atrocytomas are the most common primary malignant brain tumor (PMBT) in adults [2]. Although advances in surgery, radiation, and chemotherapy have improved length of survival in patients with an astrocytoma [3], mortality remains high which underscores the need to better understand how other factors affect the disease trajectory. Depressive symptoms are a strong prognostic indicator of poor outcome in patients with an astrocytoma [4,5] and several studies have reported an association with reduced quality of life and survival time regardless of treatment type or degree of tumor resection [6-9]. While these findings suggest that there may be common biological mechanisms underlying the influence of depressive symptoms on tumor progression and resistance to treatment, particularly for an astrocytoma [10-12], there have been no studies that have simultaneously examined these factors. Therefore, the aim of this prospective, exploratory study was to characterize depressive symptoms, other treatment-related symptoms and biological mediators that are known to influence astrocytoma growth and progression. A secondary aim was to determine whether preoperative depressive symptoms were associated with the selected biological mediators.

### Depressive Symptoms and the Tumor Microenvironment

Inflammation has long been regarded as a physiological mechanism associated with depressive symptoms. Evidence in support of this relationship has been demonstrated by studies that have observed depressive behavior following administration of proinflammatory cytokines [particularly interleukin (IL)-1, IL-6, and tumor necrosis factor-alpha (TNF-α)], either centrally (in the brain) or peripherally in animals and humans [13-15]. More specifically, IL-1, IL-6, and TNF-α have been shown to play a role in the etiology of depression by reducing the synthesis of serotonin in the brain, stimulating hypothalamic and preoptic noradrenergic neurotransmission, and inhibiting glucocorticoid receptor functioning thereby leading to hypothalamic-pituitary-adrenocortical (HPA) axis dyregulation [16].

In the central nervous system, astrocytes are the major glial cell population and act as immunocompetent cells; that is, they orchestrate local immune responses [17,18]. Part of the lethality associated with astrocytomas, as well as other gliomas, is due to the influence of an immunosuppressive tumor microenvironment which allows the tumor cells to evade immune surveillance. Cytokines play a major role in this process, along with other proteins including vascular endothelial growth factor (VEGF), matrix metallopreoteinases (MMPs) and glial fibrillary acidic protein (GFAP) [19,20] that promote angiogenesis and tumor growth [21,22]. In the tumor microenvironment, cytokines may support tumor growth, inhibit it, or play a dual function [23]. Those which have been found to suppress tumor growth include IL-2, IL-12, IL-13 and IFN (gamma, beta and alpha) while IL-4, IL-6, IL-8 and IL-10 predominantly contribute to tumor growth [24]. Thus, cytokines have been implicated in contributing to the invasiveness and vascularization of tumor cells and immunosuppression within the tumor microenvironment as well as the initiation and persistence of depressive symptoms [25,26].

To date, no studies have simultaneously characterized depressive symptoms and the local and systemic levels of these biological mediators in patients with an astrocytoma. Noting the high reported rate of depressive symptoms in patients with an astrocytoma, the detrimental effects on quality of life, and compelling data that suggests shared mechanisms underlying depressive symptoms and tumor progression [27,28], we conducted a descriptive correlational study in patients with an astrocytoma who were undergoing tumor resection.

## Results

### Sample demographics

There were a total of 22 patients with a mean age of 45.7 years (SD ± 18.09) who met inclusion criteria and were enrolled. Of these, there were 9 women (41%) and 13 men (59%) of whom 30% were diagnosed with a primary (de novo) grade III astrocytoma and 70% with a primary (de novo) grade IV astrocytoma. Lifetime history of depression was positive for 7 participants (30%) with the remaining participants reporting that they had never received a diagnosis or treatment for depression.

### Depressive symptoms

As seen in Table 1, there was a statistically significant increase in the level of depressive symptoms between baseline and the 12 month follow up assessment (*P* < .02). The mean Beck Depression Inventory version II (BDI-II) score at the time of surgery was 11.68 (SD, 1.7), indicating minimal depressive symptoms. The mean BDI-II score increased to 16.66 (SD, 2.37) at 3 months after tumor resection, slightly decreased to 15.07 (SD, 1.84) and 14.21 (SD, 1.35) at 6 and 9 months post-tumor resection respectively, and increased to 18.63 (SD, 1.48) at 12 months post-tumor resection.

**Table 1:** Symptom scores over time

	**Time**				
	**Pre-surgery (N = 22)**	**3 m post-surgery (N = 21)**	**6 m post-surgery (N = 20)**	**9 m post-surgery (N = 19)**	**12 m post-surgery (N = 19)**
BDI-II	11.68 ± 1.70	16.66 ± 2.37	15.07 ± 1.84	14.21 ± 1.35	18.63 ± 1.48
MDASI Core	2.11 ± 0.33	3.03 ± 0.27	2.20 ± 0.18	2.07 ± 0.12	2.59 ± 0.20
MDASI BT	1.25 ± 0.25	1.68 ± 0.28	1.25 ± 0.16	1.16 ± 0.17	1.85 ± 0.25
MDASI Interference	3.14 ± 0.55	4.52 ± 0.37	4.04 ± 0.42	4.35 ± 0.37	4.61 ± 0.31

Values given are mean ± standard errors. Abbreviations: *m* Months, *BDI-II* Beck Depression Inventory version II, *MDASI Core* M.D. Anderson Symptom Inventory, *MDASI BT* M.D. Anderson Symptom Inventory-Brain Tumor Module.

### Perceived Stress

Levels of perceived stress increased from baseline to the follow-up period at 3, 6, 9, and 12-months post-operatively, however, there were no significant differences between the scores over time and no significant association with depressive symptoms.

### Treatment-related symptoms

There was a statistically significant rise in the M.D. Anderson Symptom Inventory (MDASI) Core symptoms between baseline and the 3 month follow-up appointment (*P* < .003). However, there were no significant changes in the M.D. Anderson Symptom Inventory Brain Tumor Module (MDASI BT) score. The MDASI Interference score increased significantly between baseline and the 12 month follow-up assessment (*P* < .002). Baseline depressive symptoms were associated with the MDASI Core symptoms (*P* < .01) and interference scores (*P* < .002).

### Biological factors

**Figure 1:**
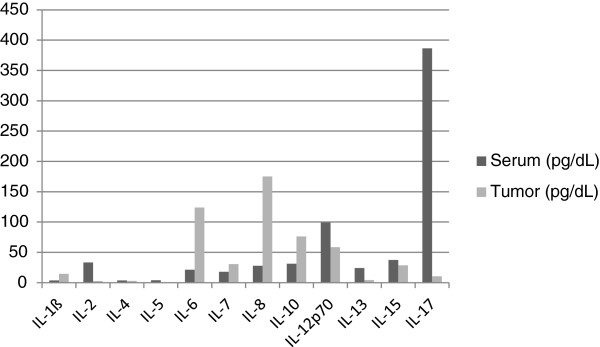
Cytokine levels in serum and brain tumor tissue.

**Figure 2:**
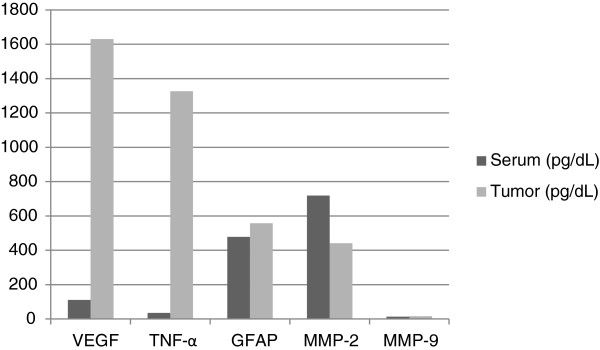
Additional biological factors in serum and tumor tissue.

The biological data from serum and brain tumor supernatant from tissue acquired at the time of surgery is presented in Figure 1 and Figure 2. Spearman correlations were performed to investigate the possibility of a relationship between biological mediators in the serum and tumor tissue, as well as between depressive symptoms and biological mediators. There were no significant associations between the serum and brain tumor levels. Of the serum cytokine levels, IL-8 was significantly negatively correlated with depressive symptoms (Spearman’ ρ = -.70, *P* < .0004) while IL-17 was the only tumor tissue cytokine associated with depressive symptoms (Spearman’ ρ = -.50, *P* < .03).

## Discussion

According to the cut-off scores of the Beck Depression Inventory version II (BDI-II), depressive symptom were minimal at baseline but showed a statistically significant increase at 12 months from baseline levels. There was also a significant rise in treatment-related symptoms at 3 months following tumor resection as well as a significant increase in symptom distress (interference score) at 12 months. These results are similar to those reported by Litofsky et al. [7] in which depressive symptoms increased over the first 6-months following tumor resection, however, in their study the MF-36 Mental Health score was used to quantify depressive symptoms and treatment-related symptoms were not measured. A systematic review of studies evaluating depression in glioma patients concluded that depressive symptoms, regardless of the measure used, tend to increase in frequency and severity over time after tumor resection [29]. The present study enhances understanding about depressive symptoms by identifying a time-related course in which there is a simultaneous rise in the severity of treatment-related symptoms at 3 months after tumor resection as well as an increase in symptom distress at 12 months.

The association between depressive symptoms and treatment-related symptoms is not surprising as previous studies have observed that depressive symptoms tend to co-occur with treatment-related symptoms, including cognitive impairment, sleep disturbances and pain in patients with a brain tumor [29,30]. Previous studies have also reported an association among depressive symptoms and decreased physical functioning, quality of life and survival in patients with brain cancer [6-9]. Mainio et al. [31] reported that increased severity of depression in patients with a brain tumor after tumor resection was associated with a lower functional status as indicated by the Karnofsky Performance Scale. Depression defined as a BDI score of 10 or greater predicted shorter survival in patients with low-grade glioma [8]. In a retrospective review of 1052 patients with malignant astrocytoma undergoing tumor resection, a preoperative clinical diagnosis of depression was a predictor of shorter survival [6]. The results of the present study demonstrate that depressive symptoms and symptom distress simultaneously increase at 12 months post-tumor resection. This finding suggests that depressive symptoms are more likely to occur when treatment-related symptoms begin to negatively impact daily functioning.

Many of the peripheral cytokines were elevated at the time of surgery . Of particular interest were the elevated levels of IL-6 and TNF-α as sustained elevations of these cytokines have been associated with “sickness behavior”, however, the levels of these cytokines were not associated with depressive symptoms in this sample. In contrast, serum levels of IL-8 were inversely associated with depressive symptoms. Although in the tumor microenvironment, IL-8 has chemotactic, tumorigenic and proangiogenic properties and has been shown to induce infiltration of tumor-associated macrophages (TAMs) [17,32], the present finding suggests a possible deregulation of peripheral neutrophil activation associated with depressive symptoms.

This study did not find an association between plasma and tumor levels of the selected cytokines. In contrast, a study by Samaras et al. [33] found that levels of IL-6 and IL-10 in astrocytic neoplasms and peripheral blood were highly correlated and that the principal IL-6 positive cell type was the neoplastic astrocyte, whereas microglial cells and macrophages were the major source of IL-10.

One of the most intriguing findings of this pilot study was the inverse relationship between depressive symptoms and brain tumor IL-17 levels. It was recently reported that IL-17 expression within tumor tissue was associated with progression-free survival in patients with an astrocytoma [34]. In that study, the authors concluded that high levels of IL-17 expression in tumor tissues may be a good prognostic marker for patients with grade IV astrocytoma. IL-17 is a CD4 T cell-derived proinflammatory cytokine that may enhance tumor suppression by signaling Th17 cells into the brain tissue. Th17 cells have enhanced migratory capabilities into the CNS because of their ability to penetrate the blood brain barrier [34]. However, due to methodological differences between studies (ie. measurement of IL-17 expression vs. level of IL-17 in tumor tissue supernatant) the significance of the present finding remains unclear and no causal inferences can be made based on the design of the study. Further investigations are necessary to examine the relationship between IL-17 in tumor tissue and depressive symptoms in a larger sample of patients and to examine the influence on patient outcomes.

### Limitations

This exploratory study was designed to explore the relationships among depressive symptoms and the selected biological factors. Most patients in the study reported minimal to moderate levels of depressive symptoms. Since the study did not include a psychological examination to differentiate participants with major depression disorder (MDD), the study findings cannot be generalized to patients with MDD. However, it can be argued that even subsyndromal MDD is associated with significant morbidity in individuals with cancer. In this context, the study findings warrant further investigation.

## Conclusions

In this sample of patients with a grade III or IV astrocytoma, depressive symptoms varied over the treatment trajectory with a significant increase at 12 months. Depressive symptoms were significantly associated with treatment-related symptoms at 3 months post-operative and symptom distress at 12 months postoperative. These findings suggest that depressive symptoms are more likely to occur when treatment-related symptoms increase or when they begin to negatively impact daily functioning. As many aspects of depressive symptoms overlap with treatment-related symptoms, including cognitive impairment and sleep disturbances, the results suggest that multi-faceted symptom management strategies may be beneficial during the postoperative period. Serum level of IL-8 and tumor tissue level of IL-17 were negatively associated with depressive symptoms, however, further research is necessary in order to determine whether these findings can be replicated in a larger sample.

## Methods

This study used a prospective, exploratory, repeated-measures design to characterize depressive symptoms, treatment-related symptoms and pro- and anti-inflammatory cytokines (as well as MMP-2, MMP-9, and GFAP) in tumor tissue and in blood at the time of tumor resection. Patient reported symptoms were also assessed at 3, 6, 9, and 12-months postoperatively. The follow-up time points were selected based on the normal course of patients after tumor resection, which includes starting chemotherapy and radiation, prognostic evaluations of the tumor response to treatment, and continued surveillance.

### Participants and procedures

This study was approved by the Institutional Review Board (IRB) of Virginia Commonwealth University, a large urban university health system in the mid-Atlantic region that served as the study site for recruitment and data collected processes. The study was conducted in accordance with the Department of Health and Human Services’ policy for protection of human research subjects.

Eligible patients were referred and screened by research collaborators in the Department of Neurological Surgery. Informed consent was obtained from each participant. Inclusion criteria included males and females 18 years or older, with clinical diagnosis of a malignant astrocytoma (Grade III or IV) who were scheduled for tumor resection. In addition, participants had to be fluent in English and without history of immune-related disease or previous cancer diagnosis. Patients were ineligible if they were unable to provide informed consent due to altered cognitive status as evidenced by a Mini-Mental Status Examination score of 23 or lower (0–23) or if they were currently taking an anxiolytic or anti-depressant medication. Subjects who enrolled in the study but were subsequently started on an anti-depressant during the postoperative period were not followed and their data was not used in the analyses. Recruitment and data collection took place between December 2010 and December 2012.

After informed consent was obtained, participants were asked to complete the study questionnaires in a private room. When a participant was found to be at risk for severe depression (≥20 on the BDI-II) or verbalized difficulty dealing with depressive symptoms, their physician was notified as explained in the informed consent. Following completion of the questionnaires, a blood specimen was collected adhering to a standard venipuncture protocol from an antecubital site on the day of surgery. Blood specimens were immediately transported on ice to the laboratory for processing.

As part of standard care for the treatment of an astrocytoma, all of the participants underwent a gross total resection, which has been associated with longer survival and improved neurological function [35]. Tissue specimens acquired during the surgery were immediately packed on ice and transported to the laboratory for processing. Surgery was followed by involved-field radiotherapy ranging from a total dose of 54 to 60 Gy and adjuvant chemotherapy with temozolomide.

### Measures

#### Demographics

Demographic and clinical characteristics of study participants (e, g., age, gender, race and ethnicity, lifetime history of depression) were recorded at baseline for each participant. Lifetime history of depression was quantified by asking the patient whether they had ever received a diagnosis or been treated for depression. If the patient answered affirmatively to either diagnosis or treatment, a positive lifetime history of depression was recorded. Age was included as a continuous variable in analyses, while gender and lifetime history of depression were dichotomous.

#### Depressive symptoms

Depressive symptoms were measured using the *Beck Depression Inventory version II* (BDI-II). The BDI-II is a 21-item self-report instrument intended to assess the existence and severity of symptoms of depression as listed in the American Psychiatric Association’s *Diagnostic and Statistical Manual of Mental Disorders Fourth Edition* (DSM-IV; 1994) [36]. There is a four-point scale for each item ranging from 0 to 3. On two items (16 and 18) there are seven options to indicate either an increase or decrease of appetite and sleep. Summing the 21 items provides a total BDI-II score that is used to provide a categorical rating of symptom severity (0 to 13 indicates minimal symptoms of depression, 14 to 19 mild symptoms, 20 to 28 moderate symptoms, and 29 to 63 severe symptoms). The total score was used in the analyses to determine the relationships between depressive symptoms and biological factors. Validity for the BDI-II has been well established in patients with a PMBT [29].

#### Perceived Stress

The Perceived Stress Scale (PSS) is the most widely used psychological instrument for measuring the perception of stress. PSS is a brief 10-item scale measuring the degree to which experiences are appraised as uncontrollable [37]. Individuals rate their responses using a 5-point Likert scale. A total score is provided by adding the responses together, with a higher score indicating a higher level of perceived stress. Internal consistency of the PSS ranges from 0.75-0.86 and test-retest reliability is 0.85 [38].

#### Treatment-related symptoms

The *M.D. Anderson Symptom Inventory-Brain Tumor Module* (MDASI-BTM) was used to measure treatment-related symptoms. The MDASI-BTM measures six symptom constructs, including affective, cognitive, focal neurological deficits, treatment-related symptoms, general/disease-related symptoms, and gastrointestinal symptoms [39]. The MDASI-BTM is a 22-item instrument consisting of 11-point scales (0–10) for each item. Participants are asked to indicate the presence and severity of each symptom in the last 24 hours, with 0 being “not present” and 10 being “as bad as you can imagine”. Items of the core symptoms (Core = 13 items) and brain tumor module symptom items (BT = 9 items) are summed and divided by the number of items in each category to provide a mean core symptom score and mean brain tumor module score. Interference (SD = 6 items) measures how much symptoms have interfered with different aspects of a patient’s life in the last 24 hours, including interference in general activity, mood, work, relations with other people, walking, and enjoyment of life. The mean of the interference items is used as a measure of overall symptom distress. Total Core, BT, and Interference scores were used in the statistical analyses. The six constructs of the MDASI-BTM have been shown to be sensitive to disease severity, treatment status, and disease progression [39].

#### Biological factors

Levels of pro- and anti-inflammatory cytokines, MMP-2, MMP-9 and GFAP were measured in serum and tumor specimens taken at the time of surgical resection. Blood samples were collected via venipuncture using a 10 mL serum separator vacutainer, immediately placed on ice and delivered to the laboratory for processing. All specimens were aliquoted immediately, frozen, and stored in a -80°C freezer until batch processing. Tumor tissue specimens were collected at the time of biopsy/resection intraoperatively as part of normal clinical procedures. No alterations in the surgery were required for this aspect of data collection. A 1.5×1.5.×0.5 cm strip of tumor specimen was cut from the tumor center and placed in a sterile container as previously described by Samaras et al. [33]. The tissue fragments were transported directly to the laboratory by a research team member. Under a reverse-flow hood each specimen was cut into three equal strips and each strip was placed in a cryovial that was snap frozen in liquid N_2_ and stored in a -80°C freezer until batch processing. At the time of analysis, each specimen was thawed under a reverse-flow hood and chopped into 1 mm^2^ pieces using crossed scalpels. The tissue fragments were immediately placed in a universal tube containing 1.5 mL of cell lysis buffer (Bio-Rad; Hercules, CA). The tissue was disrupted by drawing the samples up and down through a pipette tip 20 times followed by orbital agitation for 20 minutes at 300 rpm and 4°C. The samples were then centrifuged at 4500 *g* for 15 minutes at 4°C. Thetissue supernatant was collected for cytokine analysis using a Bio-Plex^®^ instrument following the manufacturer’s instructions.

Cytokines, GFAP, MMP-2 and MMP-9 were measured in serum and tumor supernatant using the Human 17-plex panel that includes interleukin (IL) 1beta (IL-1ß), IL-1RA, IL-2, IL-4, IL-5, IL-6, IL-7, IL-8, IL-10, IL-12p70, IL-13, IL-15, IL-17, VEGF, and TNF-α; and standardized ELISA kits (Biovendor, Candler, NC). Analysis was conducted using the Bio-Plex^®^ (Bio-Rad; Hercules, CA). All samples were run in triplicate and the data was expressed as the mean ± standard error. The assays accurately measure cytokine values in the range of 1–2,500 pg/ml, are precise (intra-assay CV <10%, interassay CV <15%), and show less than 1% cross-reactivity with other molecules.

### Statistical analyses

Means and standard deviations of the demographic data were used to describe the sample. The distributions of the biological data were graphically inspected and are presented as the mean ± standard error (SE). Student t-tests were used to evaluate differences in the patient-reported symptom scores over time and Pearson correlation coefficients were used to assess relationships between depressive symptom scores and treatment-related symptoms. Although there is controversy regarding whether to log-transform cytokine data to create normal distributions, this approach may not be ideally suited for identifying clinically important levels of the biological factors measured. Thus, Spearman rank correlations were performed for the nonparametric cytokine data. Due to the variance in serum cytokine levels, the level of significance was set at P < .001. All analyses were conducted using SigmaPlot (Systat Software, Inc., San Jose, CA). A power analysis was calculated using a study that reported significant differences in depressive symptoms among patients with a brain tumor [6] and suggested a minimum of 20 subjects were needed to obtain 90% power or greater at P < .05 significance level.

## Competing interests

The authors declare that they no competing interests.

## Authors’ contributions

ARS and PS conceived of the study and participated in its design. ARS carried out the clinical research and drafted the manuscript. PS, DEL, and DHB participated in the design of the study and coordination and helped to draft the manuscript. RKE and JS carried out the immunoassays and performed the statistical analysis. WCB participated in the design of the study, facilitated recruitment and collected tissue samples. All authors read and approved the final manuscript.
